# Exploring the progress of artificial intelligence in managing type 2 diabetes mellitus: a comprehensive review of present innovations and anticipated challenges ahead

**DOI:** 10.3389/fcdhc.2023.1316111

**Published:** 2023-12-15

**Authors:** Farwa Tahir, Muhammad Farhan

**Affiliations:** ^1^ Department of Pharmacy, Rashid Latif Medical Complex, Lahore, Pakistan; ^2^ Department of Pharmacy, University of Lahore, Islamabad, Pakistan

**Keywords:** AI, T2DM, personalized healthcare, future of diabetes care, machine learning

## Abstract

A significant worldwide health issue, Type 2 Diabetes Mellitus (T2DM) calls for creative solutions. This in-depth review examines the growing severity of T2DM and the requirement for individualized management approaches. It explores the use of artificial intelligence (AI) in the treatment of diabetes, highlighting its potential for diagnosis, customized treatment plans, and patient self-management. The paper highlights the roles played by AI applications such as expert systems, machine learning algorithms, and deep learning approaches in the identification of retinopathy, the interpretation of clinical guidelines, and prediction models. Examined are difficulties with individualized diabetes treatment, including complex technological issues and patient involvement. The review highlights the revolutionary potential of AI in the management of diabetes and calls for a balanced strategy in which AI supports clinical knowledge. It is crucial to pay attention to ethical issues, data privacy, and joint research initiatives.

## 1 Introduction

Type 2 diabetes mellitus (T2DM) is characterized by elevated blood sugar levels (hyperglycemia), resistance to insulin, and reduced insulin secretion. Its underlying causes are complex, involving both genetic factors affecting insulin function and environmental factors such as obesity (1). T2DM has a significant impact on individuals’ health and quality of life, contributing to morbidity and premature mortality. The increasing prevalence of unhealthy diets, sedentary lifestyles, high Body Mass Index (BMI), and elevated fasting plasma glucose levels have been linked to the rising rates of T2DM. Additionally, the aging global population contributes to the burden of diabetes, as it often affects older individuals. The economic cost of diabetes care is substantial, with expenses reaching 3.2 to 9.4 times the average per capita healthcare expenditure in the presence of complications. Achieving optimal control of blood glucose and other targets remains a challenge, partly due to inadequate awareness and health promotion for diabetes management (2).

### The escalating global burden of T2DM

The International Diabetes Federation estimates that the prevalence of diabetes, which was 10.5% in 2021, is projected to increase to 11.3% by 2030 and 12.2% by 2040. T2DM patients are at higher risk of organ dysfunction and failure, particularly affecting the kidneys, eyes, and nerves, leading to increased medical costs and reduced quality of life. T2DM is associated with a 15% higher risk of premature death and a roughly 20-year reduction in life expectancy. This escalating problem necessitates a deeper understanding of T2DM’s epidemiological characteristics to develop effective mitigation strategies (3).

A study indicates that the burden of T2DM has increased from 1990 to 2019, with the low-middle socio-demographic index (SDI) region experiencing the highest rise in age-standardized incidence rate, prevalence rate, mortality rate, and disability-adjusted life years due to T2DM. National data shows a positive correlation between SDIs and the increase in incidence rates (ASIR) and a negative correlation with mortality rates (ASMR). In 2019, global ASIR, age-standardized prevalence rate (ASPR), ASMR, and age-standardized disability-adjusted life years (ASDR) due to T2DM were 259.9, 5282.9, 18.5, and 801.5 per 100,000 population, respectively. Projections indicate further increases in ASIR and ASMR from 2030 to 2034 (3).

Recent estimates suggest that the number of people living with diabetes worldwide could reach 1.31 billion by 2050, a significant increase from 529 million in 2021. This surge is primarily attributed to the growing prevalence of type 2 diabetes, driven by factors like obesity and demographic shifts. By 2045, three out of four adults with diabetes are expected to reside in low-income and middle-income countries, where only a small fraction currently receive guideline-based diabetes care. Regardless of economic status, individuals facing discrimination and marginalization often bear the heaviest burden of diabetes-related consequences (4).

## 2 The imperative for personalized approaches in diabetes management

Personalized diabetes management involves tailoring clinical plans to individual patients, considering a myriad of factors. These factors encompass patient-specific, social, medical (including complications), phenotypic, biochemical, and genetic aspects, making personalized management a complex and multifaceted concept. Advances in therapeutic options for T2DM over the past decade call for a shift towards therapy tailored to a patient’s phenotype and personal characteristics. Personalized care has the potential to address two key reasons behind the ongoing morbidity and mortality associated with T2DM: suboptimal application of evidence-based therapies (due to factors like medication adherence and lifestyle changes) and the limited effectiveness of current treatments when optimally utilized (5, 6).

Personalized T2DM management now places greater emphasis on various medical and personal factors. Key medical considerations include the diabetes phenotype, biomarkers (e.g., autoantibodies, urinary C-peptide, and genetic tests), and the presence of medical comorbidities like cardiovascular or renal disease. Treatment decisions should also consider complications such as peripheral vascular disease, retinopathy, and neuropathy. Patient-related factors, such as treatment preferences, age, diabetes duration, fear of hypoglycemia, and psychosocial concerns, play an increasingly important role in developing holistic patient management plans. In the future, profiling scores incorporating clinical, biochemical, and genetic variables may further influence treatment preferences, pending additional research to establish their clinical utility (6).

Patient self-management is of paramount importance in diabetes care. Guidelines recommend that all diabetes patients engage in self-management education and behavior modification, including nutritional therapy, physical activity, appropriate medication and insulin use, hypoglycemia prevention and treatment, and psychological well-being. While continuous feedback and self-management training are recommended, time constraints during medical visits and challenges in monitoring daily blood glucose, diet, and physical activity can hinder sustained education and personalized lifestyle intervention (7, 8).

### 2.1 Self-care for patients

A patient’s insulin and blood glucose levels are impacted by physical exercise. Because of its numerous health advantages, physical activity must be given top priority in a diabetic treatment strategy. Surveys frequently ask patients with type-2 diabetes to self-report their physical activity in accordance with their treatment plan (9).

Examples of diabetes assistance self-management applications:

1- According to Maharjan (2019), a voice virtual assistant system driven by AI was created to help Native American diabetic patients manage their food intake and daily improve their understanding of health and nutrition (10).
2- A customized meal recommendation system named Ramus was created by Vaskovsky and Chvanova (2019). Ramus proposes suitable food options for diabetic patients based on their taste preferences. To deliver individualized recommendations, the system is trained utilizing data containing a variety of food impacts on patients (11).
3- A novel method was put up by Olatunji, Bolanle, Asegunoluwa, Tobore, Zedong, and Lei. It entails fusing various adaptive neuro-fuzzy inference system variants with various modalities to produce a hybrid system. the application of the MANFIS and diet guidance affective system models. Also included are details about the diagnosis model and dataset used for training and validation, which were created especially for this work, as well as some experimental data regarding the diet recommender system. Additionally, the diet guidelines’ food database was updated and retrieved (12).

### 2.2 Diabetes personalized medicine

There is considerable interest in employing unique molecular indicators to guide patient-specific therapy of diabetes as a result of significant breakthroughs in defining human gene sequences. The ability to analyze millions of genes, proteins, and metabolites thanks to AI developments in genetics, genomics, proteomics, and metabolomics has opened up new options for finding genetic variables and gene products linked to various diabetes subtypes (9).

An illustration is the novel diabetic treatment recommendation system provided by Sang, Jongyoul, Su, Seungyeon, and Jeonghoon, which blends the concepts of contextual bandits and reinforcement learning. The model was created using electronic health records from a South Korean database that receives one million patient records’ worth of updates per year (13).

### 2.3 Diabetes individualized therapy approaches

Artificial intelligence (AI) is a practical technology that can help in the treatment of diabetes. Five AI strategies that can help with diabetes treatment are as follows:

#### 2.3.1 Prediction of blood glucose

Methods based on data that are relevant to blood glucose utilize the gathering of data and finding the information concealed in the data to forecast future blood glucose levels (9). Contrary to systems that mimic the human physiology of the glucose-insulin regulation system, data-driven glucose prediction does not require comprehension of the physiology of diabetes. These techniques primarily rely on collected data and extract hidden information from it to project future blood sugar levels (14).

#### 2.3.2 Detection of hyperglycemia

Machine learning (ML) approaches can be used to solve the regression problem of blood glucose prediction. As a classification problem, detecting hypo- or hyperglycemia can be understood as determining whether a given input dataset will likely result in a hypo- or hyperglycemic episode (15). According to Sudharsan (16), Hypoglycemic and hyperglycemic events in people with type 2 diabetes can be accurately diagnosed even with sporadic blood glucose readings collected once or twice a day through self-monitoring (SMBG). The prediction of hypoglycemia occurrences over the next 24 hours obtained 92% sensitivity and 70% specificity after the model was trained on data spanning roughly 10 weeks. Although the forecast range was restricted to the time when hypoglycemia occurred, the specificity of the prediction increased to 90% by including drug information from the previous few days.

#### 2.3.3 Detecting glycaemic variability

Identification of glycemic variability: Variations in blood sugar levels, which increase the risk of hypo- and hyperglycemia, are a sign of insufficient diabetes controlled (17). Marin et al. (18) used support vector regression (SVR) techniques and multilayer perceptrons (MPs) to divide the consensus indicator of perceived glycaemic variability (CPGV) into four classes of CV (low, borderline, high, and very high) based on 250 24-hour continuous glucose monitoring (CGM) maps. By employing 10-fold cross-validation and hand scoring, the CPGV metric achieved 90.1% accuracy, 97.0% sensitivity, and 74.1% specificity. SVR fared better than MPs, as well as MAGE or SD, among other metrics. Hua, Ilya, and Wei used a framework for personalized RL (Reinforcement Learning) in the context of type 2 diabetes, which resulted in significant improvements in glycemia, blood pressure, and CVD risk outcomes while also demonstrating a high level of agreement with the recommendations made by clinicians (12).

#### 2.3.4 Insulin controller therapy

As a model-free method that does not require a mathematical model of the relationship between glucose and insulin, machine learning can be used to regulate blood glucose levels (19). Zita (20) used the multilayer feedforward neural network (LM-NN) and polynomial network (PN) as two different artificial neural network (ANN) models as controllers for insulin dose titration. Using a dataset of 30,000 blood glucose measurements from 70 distinct patients, the simulations were run, and LM-NN outperformed PN. The authors claim that LMNN may be used as a model-free insulin controller.

#### 2.3.5 Pharmacogenetics

In the area of type 2 diabetes, pharmacogenetics offers a chance to provide tailored medication. Certain forms of monogenic diabetes are already treated with personalized therapy. Given the tremendous progress made in recent decades, “personalized diabetology” is projected to be used more frequently in T2DM in the years to come. Genome-wide association studies (GWAS), candidate gene research, extensive genotyping surveys, and genetic engineering have all produced intriguing findings that may influence therapeutic practice. For some monogenic forms of diabetes, pharmacogenetic research has begun to realize the possibility of individualized diabetes treatment. Also helpful for personalized medicine is the field of “miRNA pharmacogenomics,” which investigates polymorphisms in miRNA-regulated pathways and their associations with drug response (21).

## 3 AI applications in T2DM diagnosis

“AI is defined as a field within computer science dedicated to developing systems or methods capable of analyzing information and handling complexity across various applications”.

The incorporation of AI into the management of diabetes is not only practical but also desirable, providing efficient data handling and the development of tools and devices to enhance care. To ensure the safety of AI applications in healthcare, it is advisable to implement secure designs, safety reserves, and procedural safeguards, identifying and mitigating uncertainties in potential technical systems (22).

Artificial intelligence (AI) has been applied in various medical fields for many purposes. Actually, AI is concerned with the computational understanding of what is commonly called intelligent behavior with the creation of artifacts that exhibit such behavior” (23). Artificial intelligence (AI) has found applications in various medical fields, including diabetes management. AI encompasses the computational understanding of intelligent behavior, creating artifacts that exhibit such behavior.

### 3.1 Expert systems in medicine

In medicine, expert systems (ES) are commonly used AI tools. ES capture expert knowledge, facts, and reasoning techniques to assist healthcare providers in decision support and problem-solving tasks. They manage data to draw reasoned conclusions, with applications in image interpretation, diagnostic support, and alarm generation, among others.

### 3.2 RBR, CBR & FL

Rule-based reasoning (RBR), case-based reasoning (CBR), and fuzzy systems are common ES utilized in diabetes-related domains. RBR involves encoding knowledge in “if-then” statements to mimic expert decision-making. CBR identifies solutions to new problems by adapting solutions from similar past cases (23).

Fuzzy logic (FL) systems handle ambiguous terms by assigning degrees of membership to categories, enabling more nuanced decision-making.

#### 3.2.1 CBR based decision support systems for patients

Decision support systems using AI assist patients in tracking food intake and improving nutritional knowledge. They provide personalized food recommendations based on taste preferences and suggest appropriate dietary choices. AI-powered voice assistants help Native American diabetes patients manage their health effectively (24).

#### 3.2.2 Closed-loop systems

Closed-loop systems based on FL algorithms have shown promise for blood glucose control. They utilize continuous glucose monitoring data and AI to predict and adjust insulin doses, reducing the risk of hypoglycemia and hyperglycemia. In silico clinical trials that simulate closed-loop systems are also under development to inform their potential benefits and challenges (25, 26).

### 3.3 Machine learning

Machine learning (ML) algorithms, a subset of AI, learn over time without explicit programming and are often used for data classification. ML methods, including decision trees (DT), artificial neural networks (ANN), genetic algorithms (GA), and support vector machines (SVM), have been successfully applied in diabetes management.

ANNs, inspired by the human brain, consist of interconnected neurons that process input data to generate outputs. GA simulates natural selection to optimize solutions for specific problems, while SVM defines hyperplanes to classify data points for binary or multiclass problems. These AI methodologies have been employed in various diabetes-related tasks, from screening to blood glucose classification. Deep learning, an evolution of ANNs, utilizes hierarchical layers to process input data, making it especially powerful for tasks such as image analysis and natural language processing. These deep neural networks have been used to identify diabetic retinopathy and diabetic macular edema from retinal images with high sensitivity and specificity (23, 27, 28).

#### 3.3.1 Using ANN retinopathy detection

AI has been instrumental in diabetic retinopathy (DR) detection. Deep learning algorithms can analyze retinal images to identify DR with high accuracy, allowing for early intervention and prevention of vision loss (29).

### 3.4 (CIGs) computer interpretable guidelines

Computer-interpretable guidelines (CIGs) are AI-based systems that transform medical guidelines into a format that computers can understand and use for decision support. CIGs help physicians make evidence-based decisions by matching patient data to clinical guidelines (30).

### 3.5 Prediction and risk stratification models

These models in T2DM use AI to analyze large datasets to forecast disease progression, complications, and outcomes. These models can identify patients at high risk for adverse events, enabling targeted interventions and better resource allocation (31).

### 3.6 Application of AI in early diagnosis and patient management

AI empowers patients by delivering information and digital solutions that have a profound impact on healthcare systems. It shapes patient comorbidities, behaviors, the duration of healthcare facility visits, and the need for frequent travel and interaction with healthcare providers. The integration of AI has optimized patient flow within hospitals and streamlined the transfer of patients between different hospital departments.

Engaging in online diabetes communities and support groups provides patients with an avenue to connect and draw insights from the experiences of others. This collaborative learning approach, involving both patients and caregivers, positively shapes desired outcomes and contributes to the overall well-being of patients.

Leveraging AI for the early detection of diabetic retinopathy proves to be a cost-effective alternative, effectively mitigating ophthalmic complications and preventable blindness associated with diabetes. Continuous glucose monitors (CGMs) have the potential to reduce healthcare costs related to diabetes. Moreover, the adoption of image-based screening for retinal changes and diabetic foot ulcers can eliminate delays in referrals for specialized care, enhancing the quality of life through prompt and timely interventions (22).

#### 3.5.1 Anticipating diabetes onset

In the assessment of adults’ vulnerability to type 2 diabetes, British researchers systematically examined 94 existing models and scoring criteria for type 2 diabetes risk, employing a combination of quantitative and qualitative approaches. They meticulously chose specific models and scoring methods for practical use, subjecting them to rigorous testing to validate their effectiveness. The experimental outcomes demonstrated the robustness of a substantial portion of the risk assessment approaches, affirming their efficacy across diverse patient groups (32). Another investigation by Mani et al. (33) utilized various machine learning algorithms in conjunction with electronic medical record data from 2,000 patients with type 2 diabetes to predict the likelihood of diabetes. The results revealed that the area under the curve (AUC) values for predictions at 6 months and 1 year were <0.8, highlighting the potential of employing electronic medical record data for automated diabetes prediction and identifying individuals at an elevated risk of developing the condition. Fu et al. devised a risk assessment model for postprandial hyperglycemia with the aim of pinpointing individuals at high risk for this condition, ultimately reducing the number of people necessitating an oral glucose tolerance test (OGTT) (34, 35).

## 4 Challenges in personalizing diabetes therapy

These primarily revolve around technical implementation rather than medical aspects. Key hurdles include:

### 4.1 Challenge 1

The complexity of the human insulin system and the evolving nature of AI technology necessitate collaboration with diabetes specialists. Sole reliance on AI for personalized treatment might lead to complications.

### 4.2 Challenge 2

Diabetes treatment is intricate, varying widely among patients due to factors like physical activity and overall health. Personalized therapy optimization becomes challenging, especially considering the array of treatment options available for type 2 diabetes patients.

### 4.3 Challenge 3

Although numerous smartphone apps calculate insulin doses, using unauthorized medical software risks unsafe dosages. Ensuring patients avoid such applications is crucial.

### 4.4 Challenge 4

Patient engagement is vital, requiring regular documentation of factors like blood glucose levels. However, elderly or unmotivated patients find this overwhelming. Creating minimally intrusive, interactive treatment aids is essential for managing their condition without burdening them further.

### 4.5 Challenge 5

Machine learning algorithms predicting blood glucose values rely on data quality. Data scarcity, especially concerning critical situations like hypoglycemia, can lead to unsatisfactory predictive results, impacting patient safety (9).

## 5 Conclusion

AI technologies hold significant promise in improving various aspects of diabetes management, from diagnosis to personalized treatment and monitoring. As our understanding of diabetes deepens and AI capabilities continue to evolve, we can expect to see more innovative applications in this field. Machine learning algorithms have primarily been employed to categorize individuals at risk of diabetes into pre-diabetes, diabetes, and advanced diabetes based on their HbA1c levels. These algorithms are utilized to assess risk factors for Type 2 Diabetes Mellitus (T2DM) across diverse populations, identifying patient groups that may necessitate increased attention for the prevention of (1) disease onset, (2) progression to more severe stages, and (3) the development of complications. While AI can enhance decision support for healthcare providers and empower patients in self-management, it is essential to ensure the ethical use of AI and address issues related to data privacy, bias, and transparency. Moreover, AI should be viewed as a complementary tool in diabetes care, working in tandem with clinical expertise to achieve the best outcomes for patients. Continued research and collaboration between healthcare professionals, researchers, and AI experts are critical to harnessing the full potential of AI in diabetes management and improving the lives of individuals living with T2DM.

## Author contributions

FT: Conceptualization, Writing – original draft, Writing – review & editing. MF: Visualization, Writing – review & editing.
